# The rising income gradient in life expectancy in Sweden over six decades

**DOI:** 10.1073/pnas.2418145122

**Published:** 2025-03-31

**Authors:** Johannes Hagen, Lisa Laun, Charlotte Lucke, Mårten Palme

**Affiliations:** ^a^Jönköping International Business School, Jönköping University, Jönköping 553 18, Sweden; ^b^Institute for Evaluation of Labour Market and Education Policy (IFAU), Uppsala 751 20, Sweden; ^c^Department of Economics, Stockholm University, Stockholm 106 91, Sweden

**Keywords:** health inequality, life expectancy, health disparities, income inequality

## Abstract

This study examines the long-term association between income and life expectancy in Sweden between 1960 and 2021. The study is based on register data that include all Swedish permanent residents aged 40 y and older. The results show that the gap in life expectancy between the top and bottom income percentiles widened substantially: For men, it increased from 3.5 y in the 1960s to 10.9 y by the 2010s, and for women, from 3.8 y in the 1970s to 8.6 y by the 2010s. Despite a reduction in income inequality and an expansion of social spending from the 1960s to the 1990s, health inequality continuously increased over the period under study. The changes of the relation between real income and life expectancy, the so-called Preston curve, reveal a much faster improvement in life expectancy in the upper half of the income distribution than suggested by the cross-sectional relation between income and life expectancy. Analysis of causes of death identified circulatory diseases as the main contributor to improved longevity, while cancer contributed more to the increased gap in life expectancy for women and equally for men. Finally, analysis of the change in the income gradient in avoidable causes of death showed the strongest contribution of preventable causes, both for men and women.

## Significance

This study reveals that the income gradient in life expectancy in Sweden has steadily increased since the 1960s, despite a reduction in income inequality until 1990. This challenges the “absolute income hypothesis”—the notion that economic resources per se affect life expectancy and that increasing income inequality directly drives health disparities. Instead, a “third factor” appears to be associated with both income and life expectancy, leading to greater gains in life expectancy among higher income groups. These gains are evident in both preventable and treatable disease mortality and appear more strongly for preventable causes, suggesting that higher-income individuals are more rapidly adopting healthier lifestyles. This finding highlights the need to consider factors beyond economic resources in addressing health inequalities.

Numerous studies, with backgrounds in different social sciences, have documented a positive relationship between measures of socioeconomic status (SES) and life expectancy (1–7). Several influential studies have explored the income gradient in mortality in different countries (8–15) and, with a notable exception (16), have also suggested that this gradient has increased in recent years. However, the focus of these studies leaves significant aspects of the long-term evolution of the income gradient in life expectancy, the relationship between income inequalities and health inequalities, and the influence of income on mortality unexplored.

Sweden provides an interesting case for empirically studying the long-term relationship between income and life expectancy. The country witnessed decreasing income inequality from the early 1960s to the 1990s and an increase thereafter. Furthermore, Sweden’s tax-funded health and welfare systems provided enhanced support to those at the lower end of the income distribution (17). The availability of administrative income data, linked with mortality data including causes of death from 1960 to 2021, enables a comprehensive analysis of the gradient’s evolution over six decades.

The four aims of this study are to 1) show the long-term development of the slope and the shape of the income gradient in mortality; 2) examine the association between income inequality and the income gradient in life expectancy; 3) assess the relationship between real income and life expectancy over time; and 4) decompose the income-related differences in life expectancy into causes of death before age 75: first, based on the main categories and, second, based on preventable and treatable causes.

## 1. Results

The data and empirical approach are described in *Materials and Methods*. The main study population consisted of close to 9.4 million persons aged 40 y or older and almost 214 million person-year observations between 1960 and 2021, accounting for almost 3.9 million observed deaths. Individuals with missing, negative, and zero income, as well as the lowest 3% of the income distribution, were excluded. Individuals received income ranks based on equivalised household income by gender, age, and year, from two years (three years in 1969 due to missing data) prior to calculating within-group mortality rates. The study used income data during 1960–2019 and mortality data during 1962–2021. Due to the two-year lag of income relative to mortality and incomplete income data for women until 1968 (*Materials and Methods*), ranks and thereby inequality measures were calculated from 1962 to 2021 for men and from 1970 to 2021 for women.

### 1.1. Trends in the Income Gradient in Mortality.

**Fig. 1.**
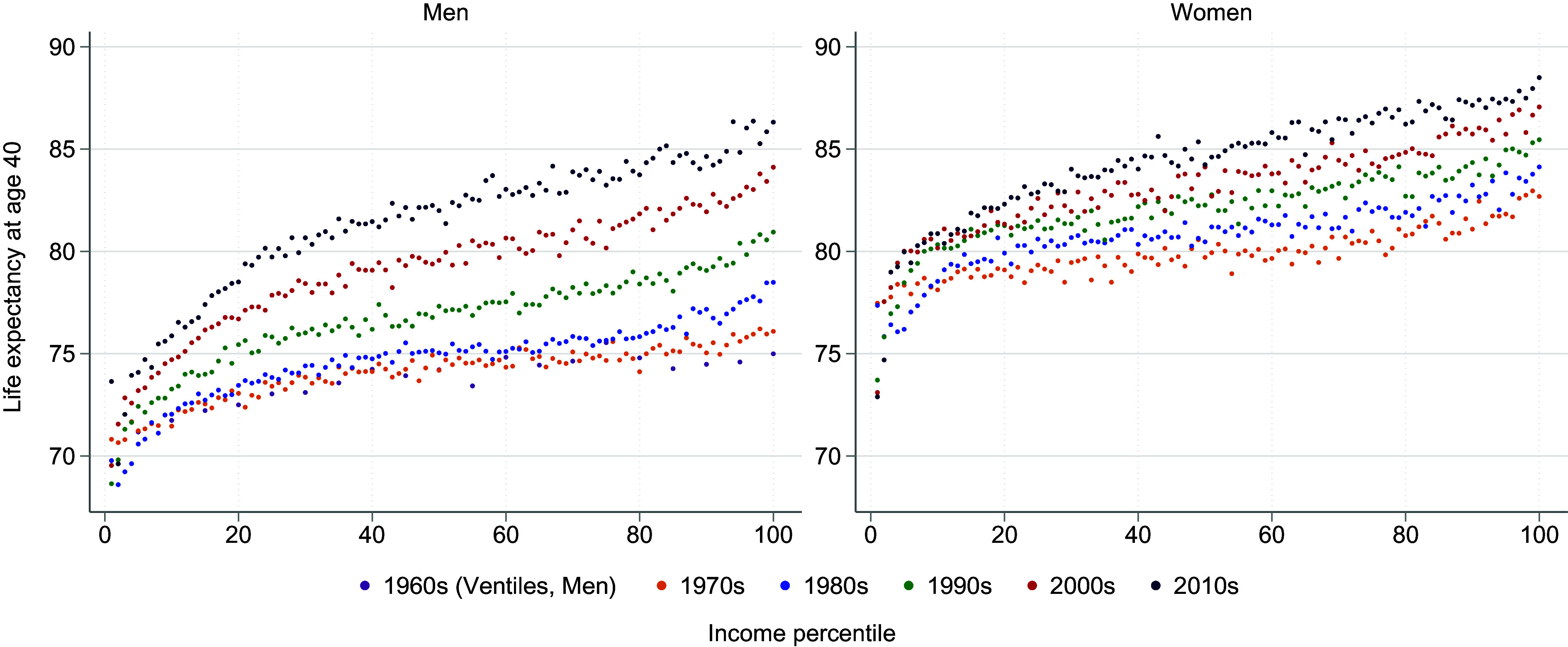
The relationship between life expectancy and income rank in Sweden. Average life expectancy at age 40 for each percentile of the income distribution by decade, for men and women. Because of limited data, distributions for the 1960s are presented by ventiles and for men only. The data cover 1962–2019 for men and 1970–2019 for women.

Fig. 1 illustrates life expectancy at 40 y of age by percentiles of the income distribution for each studied decade, highlighting three key observations. First, it underscores the established concave link between income and life expectancy noted in earlier research (18–20). Second, upward shifts in life expectancy in all income brackets over all decades suggest general health improvements. Third, the progressively steeper slope of this income-life expectancy gradient over time points to a widening gap in inequality in life expectancy with larger gains at the higher end of the income distribution.

Table 1 presents the estimated life expectancy difference in years between the top and bottom percentiles of the income distribution. For men, the difference grew from 3.5 y in the 1960s to 10.9 y in the 2010s. For women, changes in the income gradient were less pronounced. The gap in life expectancy between women in the top and bottom percentiles widened from 3.8 to 8.6 y between the 1970s and 2010s.

**Table 1.** Life expectancy gap in years between top and bottom percentiles of the income distribution by decade, for men and women

		Decade					
		1960s (Men)	1970s	1980s	1990s	2000s	2010s
Men	Coefficient	3.47	4.17	6.08	7.57	9.43	10.92
	95% C.I.	[2.85, 4.09]	[3.83, 4.52]	[5.56, 6.60]	[7.00, 8.13]	[8.76, 10.09]	[10.03, 11.82]
Women	Coefficient	–	3.76	5.16	5.84	6.94	8.56
	95% C.I.		[3.43, 4.08]	[4.66, 5.66]	[5.23, 6.45]	[6.34, 7.55]	[7.78, 9.34]

Slope coefficients from a linear regression of percentile life expectancy on the corresponding percentile of the income distribution, as presented in Fig. 1, multiplied by 100 to achieve the difference in years between top and bottom percentiles. CIs at the 95% significance level in square brackets. Because of limited data, estimates for the 1960s are presented for men only.

### 1.2. The Association Between Income Inequality, Social Spending, and the Income Gradient in Life Expectancy.

**Fig. 2.**
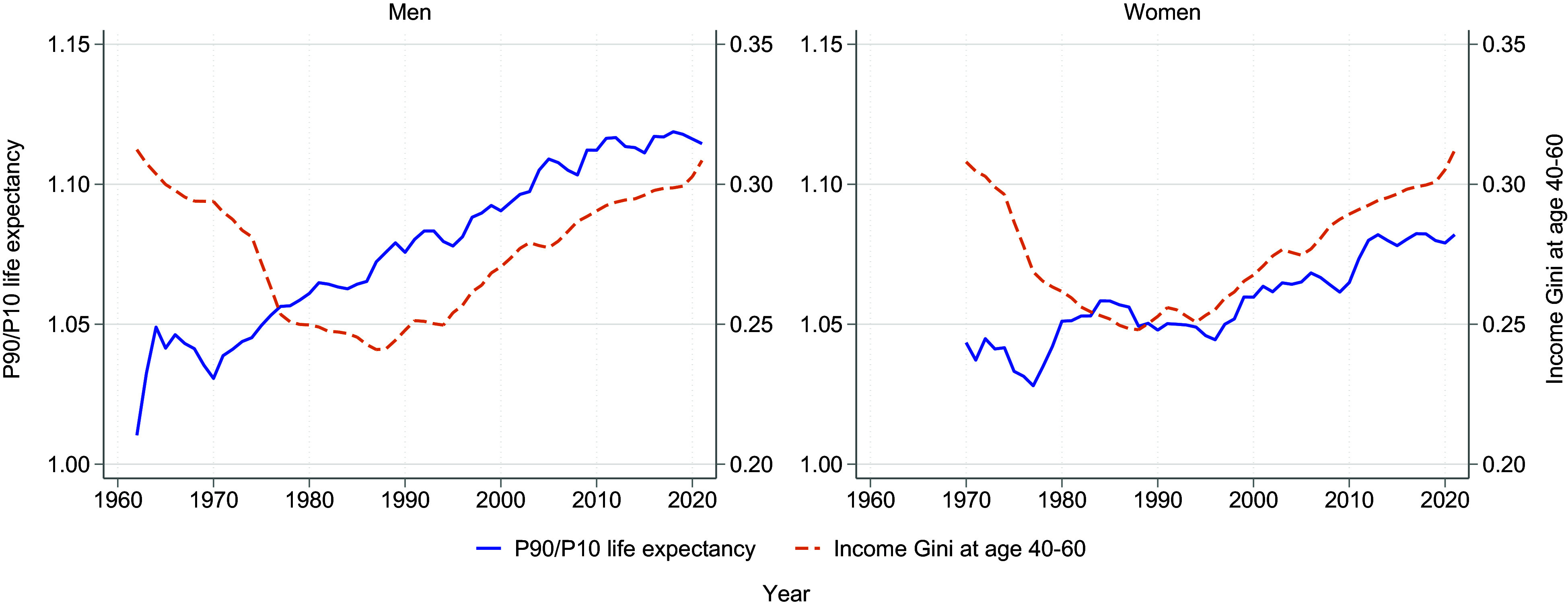
Inequality in income and life expectancy, 1962–2021 for men and 1970–2021 for women. Inequality in income is measured by calculating the Gini coefficient for income at ages 40 to 60. Inequality in life expectancy is measured by the ratio of life expectancy at age 40 between the 90th and 10th percentile (P90/P10) of the income distribution. 3-y moving averages for both series.

Fig. 2 shows the development of income inequality and the income gradient in life expectancy from 1962 to 2021 for men and from 1970 to 2021 for women. Income inequality was measured by the Gini coefficient (21) of the gender-specific income distribution, adjusted for cohort size, while the income gradient in life expectancy used ratios between the 90th and 10th percentiles (P90/P10) of the income distribution. Income data were taken from ages 40 to 60, where age 40 correlates strongly with lifetime earnings (22).

Fig. 2 shows a very salient result: While the income gradient in life expectancy increased continuously for both men and women during the entire period under study, income inequality decreased markedly for both gender groups until the early 1990s and increased only after that. These results do not provide empirical support for a long-term positive association between income inequality and the income gradient in longevity.

The expansion of the welfare state could be expected to reduce health inequalities by redistributing to low-income individuals. However, the association goes in the other direction, with increasing social spending until the 1990s coinciding with an increased income gradient in life expectancy (*SI Appendix*, Fig. S5). Social spending was measured as a share of Gross National Product (GDP), derived from Organisation for Economic Co-operation and Development (OECD) data, including health spending, government transfers, and pensions.

### 1.3. The Association Between Life Expectancy and Real Income.

**Fig. 3.**
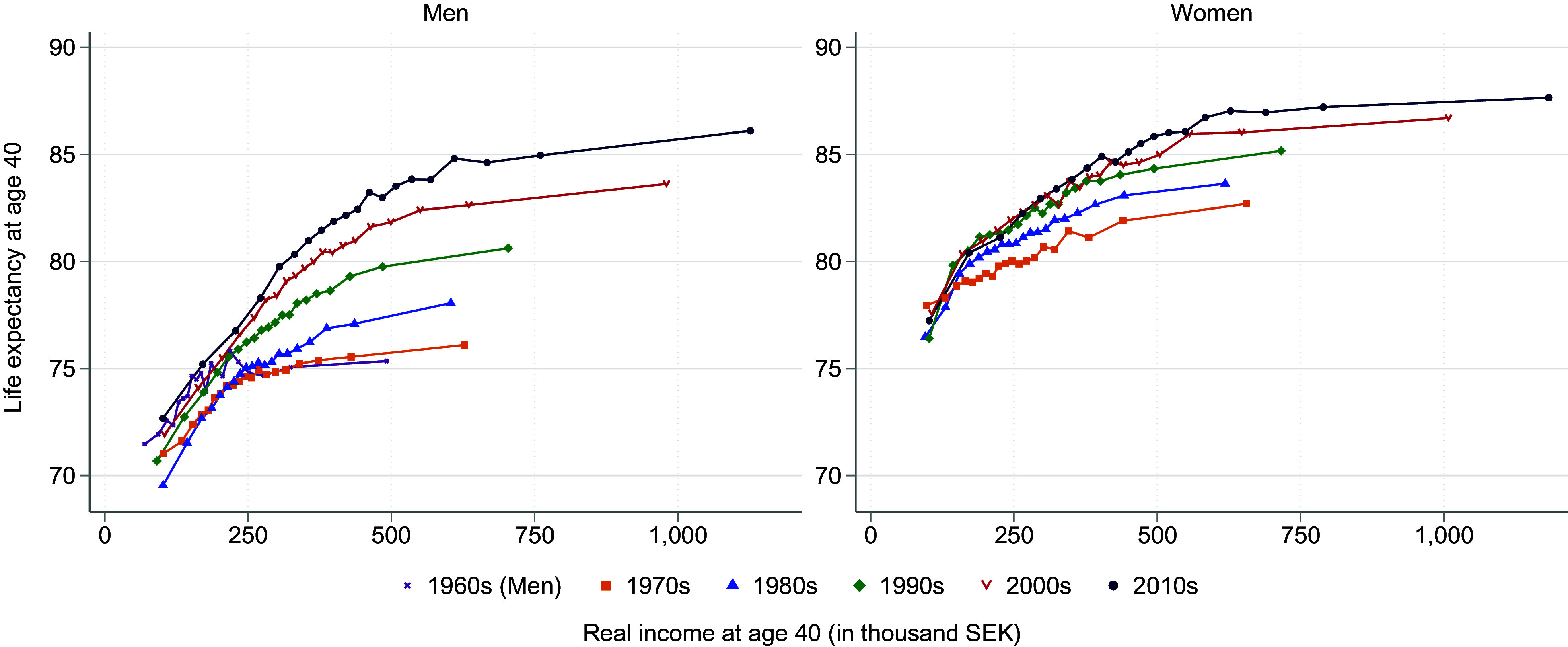
Life expectancy by income. Average life expectancy at age 40 and average annual real income (2018 SEK) for each ventile of the income distribution, by decade, for men and women.

At the cross-country level, the association between GDP per capita and life expectancy is known as the Preston curve (23). Fig. 3 shows the Preston curves for Sweden over the six decades from the 1960s to the 2010s, showing life expectancy at age 40 by ventile average income, adjusted for Consumer Price Index (CPI), for both genders. The interpretation of changes in Preston curves over time is such that movements along the cross-sectional relation correspond to improvements predicted by the cross-sectional association between income and life expectancy, while “shifts” in the curve correspond to changes exogenous to the relation depicted by the curve.

The most salient result revealed in Fig. 3 is the “shifts” in the Preston curve at the upper end of the distribution, in particular for men, reflecting the impact of external (“third”) factors, such as improvements in medical technology or changes in lifestyles related to health. The increasingly concave relationship, particularly flat at higher income levels, suggests a diminishing importance of income alone in life expectancy.

### 1.4. Contributions from Different Causes of Death.

**Fig. 4.**
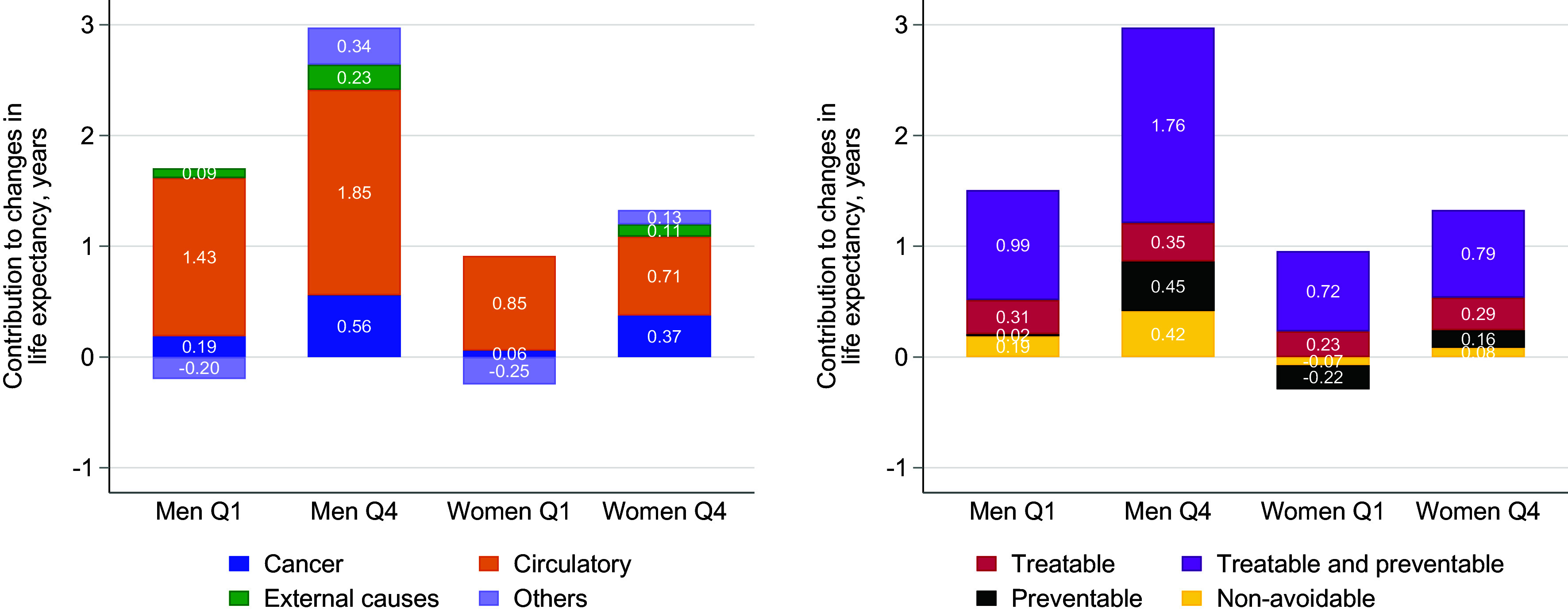
Contribution of each cause of death to changes in life expectancy due to reduced premature mortality (deaths before age 75), in years. Values smaller than 0.01 in absolute value were not displayed separately and instead added to Other diseases (for exact numbers see *SI Appendix*, Table S3). Deaths from nervous system diseases were included in the circulatory system diseases category due to the reclassification of vascular lesions from ICD-7 to ICD-8 (see *SI Appendix*, Table S1 for further details).

Fig. 4 shows the number of years of increase in life expectancy from lower mortality in premature deaths (deaths before age 75) in the first (Q1) and fourth (Q4) quartiles of the income distribution by different causes of death during the observation period. The method was used in previous research (24) and consists of scaling changes in mortality rates by the general gain in life expectancy due to reductions in premature mortality (see *Materials and Methods* for details). The total gain in life expectancy from reduced premature mortality was 1.51 y for men in Q1 and 2.97 y in Q4. Among women, the corresponding gains were 0.67 y in Q1 and 1.33 y in Q4. Fig. 4 divides these gains into contributions by different causes. The contributions of different causes to the total differential life expectancy gain between Q1 and Q4 over the observation period, which was 1.47 y for men and 0.66 y for women, are also compared (see *SI Appendix*, Table S3 for exact numbers).

**Fig. 5.**
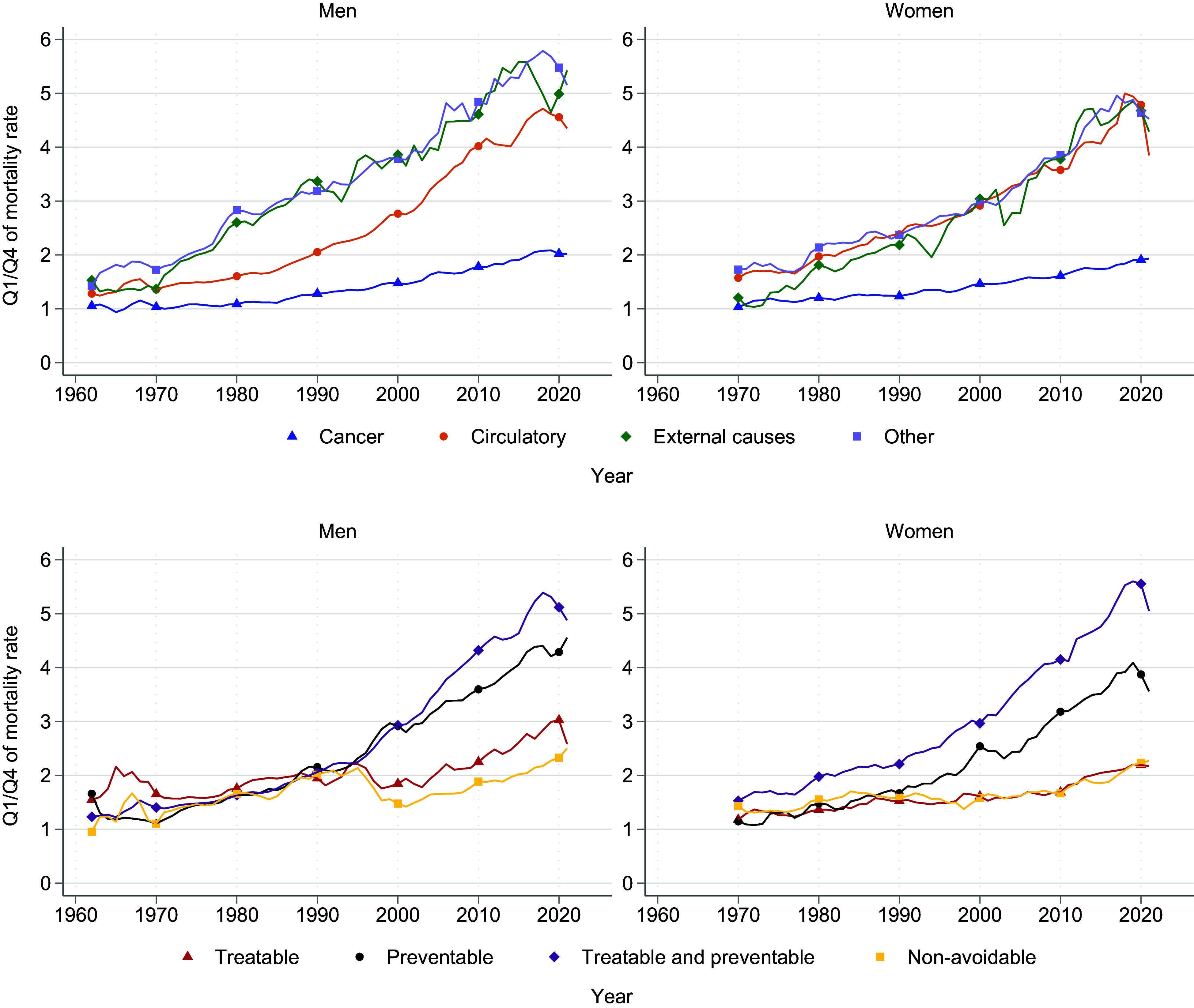
Changes in mortality ratios between the first and the fourth quartiles of the income distribution, by cause of death, during 1962–2021 for men and 1970–2021 for women. The *Top* row shows the Q1/Q4 ratio by main ICD Chapter for cancer, circulatory diseases, external causes, and other causes for men and women. The *Bottom* row shows the Q1/Q4 ratio for treatable and preventable causes of death according to the OECD/Eurostat classification for men and women. 3-y moving averages for all series.

To examine changes over time in more detail, Fig. 5 presents the Q1/Q4 ratios of mortality before age 75 by different causes of death, from 1962 to 2021 for men and from 1970 to 2021 for women. An upward trend in the ratio indicates growing income-related disparities in mortality.

The results in Figs. 4 and 5 are first presented by the main causes of death, separating between Circulatory diseases, Cancers, External causes, and Other causes. The results are then presented by avoidability, separating between Treatable, Preventable, both Treatable and preventable, or Nonavoidable causes (see *Materials and Methods* for details). When interpreting the gains in life expectancy from Cancer, it is important to bear in mind that the incidence of dying from Cancer increases due to the large gains in life expectancy from decreased circulatory deaths. The reported estimates of life expectancy gains from Cancer may therefore underestimate the true improvements in cancer care (25).

#### 1.4.1. Main causes of death by ICD chapter.

The *Left* panel of Fig. 4 shows that circulatory diseases contributed 1.43 y to the additional life expectancy in Q1 and 1.85 y in Q4 among men, making them the largest contributor in both groups. It is also the largest contributor for women, with gains of 0.85 y in Q1 and 0.71 y in Q4. The second-largest improvement came from Cancers. The highest gain, 0.56 y, was observed among males in Q4, while males in Q1 experienced a gain of 0.19 y. Among women, Cancers contributed 0.37 y in Q4 but only 0.06 y in Q1.

Of the 1.47-y difference in additional life expectancy between Q1 and Q4 for men, Circulatory diseases accounted for 0.43 y (29%) (*SI Appendix*, Table S3). For women, Circulatory diseases reduced the difference by 0.14 y (−21%) between Q1 and Q4. The corresponding contributions for Cancer-related mortality are 0.37 y (25%) for men and 0.32 y (48%) for women. Thus, while reductions in premature mortality from circulatory diseases accounted for the largest overall gains in life expectancy, improvements in cancer-related mortality showed an almost equally pronounced income gradient for men and an even steeper gradient for women.

Reductions in mortality due to External causes contributed 0.09 and 0.23 y of additional life expectancy among men in Q1 and Q4, respectively, and 0.00 and 0.11 y among women. This means that external causes contributed 9% and 18% of the difference in additional life expectancy between Q1 and Q4, respectively. While there is a clear income gradient, with greater gains observed in the upper part of the income distribution, the relatively low incidence of external causes compared to circulatory diseases and cancer limits their overall contribution to mortality differences.

Finally, for Other causes, the corresponding changes in life expectancy were −0.20 y for men in Q1 and 0.34 y in Q4, while for women, the changes were −0.24 y in Q1 and 0.13 y in Q4. This constitutes a significant portion of the total differential life expectancy gain between Q1 and Q4, amounting to 36% for men and 56% for women.

Turning to the evolution of the Q1/Q4 mortality ratios in Fig. 5, the *Upper* panels illustrate that the ratio for circulatory diseases tripled over the study period. The consistently upward trend in the ratios, beginning a decade later for men, indicates a steady rise in mortality inequality from circulatory diseases over time, with the disparity becoming increasingly pronounced toward the end of the period. When interpreting this result alongside the absolute gains in Fig. 4, it is important to note the substantial decline in circulatory disease mortality, particularly in Q4 (*SI Appendix*, Fig. S6). As rates in Q4 reach low levels, even small differences between Q1 and Q4 are amplified in the Q1/Q4 ratio, increasing relative inequality despite similar absolute gains in life expectancy.

For Cancers, the ratios in Fig. 5 were initially around 1, indicating that there were no income-related disparities in the initial period. However, they began to increase in the 1980s, reaching a value of 2, indicating a 100 percent higher cancer mortality in Q1 compared to Q4, by the end of the period under study. As shown in Fig. 4, this increase is driven by substantial life expectancy improvements among higher-income groups, while lower-income groups experienced only very small gains during this time.

#### 1.4.2. Avoidable mortality.

The *Right* panel of Fig. 4 shows the contributions of Treatable, Preventable, combined Treatable and preventable mortality, as well as Nonavoidable causes, to gains in life expectancy for Q1 and Q4 for men and women. The greatest gain in life expectancy from mortality before age 75, in all subgroups studied, was attributed to the combined category of treatable and preventable causes of death. However, the largest difference in life expectancy gains was attributed to the pure preventable category. For this category, there were much larger improvements in Q4 compared to Q1 for both men and women. For men, 53% of the difference in the increase in life expectancy in Q4 compared to Q1 was attributed to the combined category of treatable and preventable and 29% to the pure preventable category (*SI Appendix*, Table S3). For women, this figure was 10% and 57%, respectively. The improvement in life expectancy as a result of reduced mortality from Treatable conditions was almost equally shared between Q1 and Q4 for men and women.

The *Bottom* row of Fig. 5 illustrates the evolution of inequality in mortality by avoidability, using the Q1/Q4 mortality ratio. The Q1/Q4 ratio of diseases classified as Treatable and preventable, as well as purely Preventable, increased dramatically, indicating substantial increases in inequality, particularly among men. The corresponding ratios for Treatable mortality, while also increasing, followed a more moderate trend, especially for men, suggesting a slower growth in inequality for treatable causes. Nonavoidable causes exhibited relatively stable ratios over time, with more limited changes in income-related disparities.

## 2. Discussion

This paper extends the previous literature on the historical development of the income gradient in life expectancy; the relationship between income inequality and health inequality; the evolution of the relationship between real income and mortality in different parts of the income distribution; as well as how different causes of death have contributed to the change.

Nonparametric estimates of the historical development of the income gradient in health in Sweden extend previous research by covering the entire income distribution at the percentile level over a much longer period than earlier studies, which often focus on shorter time periods (8, 9, 12–14, 16), income quintiles (10, 26, 27), specific parts of the population (1, 28), or measuring the education gradient (3).

Previous studies have investigated the relationship between income inequality and life expectancy (18, 29–31), as well as the relationship between income inequality and the income gradient in life expectancy using cross-sectional data (8). A contribution of this paper is that it examines the relationship between income inequality and the income gradient in mortality over time, spanning almost 60 y and including periods of both rising and declining income inequality.

This paper also examines the relationship between real household income and life expectancy, known as the Preston curve (23), over the six decades covered by the data. Previous studies have estimated this relationship either as a cross-section between different countries or within a single country between income groups for two periods close in time (16, 23).

Finally, the analysis of the causes of death underlying the increase in the income gradient extends the previous literature (10, 12, 26, 32) by examining a longer period of time and explicitly utilizing the OECD/Eurostat ICD classification of avoidable deaths, distinguishing between mortality due to preventable and treatable causes.

Our results demonstrate that the income gradient in life expectancy is a relatively new phenomenon in Sweden. In the 1960s, it was virtually nonexistent. Since then, it has steadily emerged and increased. In particular, this trend appears to be unrelated to the evolution of income inequality. Even during the period from 1960 to 1990, marked by a move toward a more equal income distribution, the income gradient in life expectancy grew. This suggests that a strong association between individual income and mortality, as implied by the absolute income hypothesis (18, 19), is unlikely. In this sense, our findings support previous research that indicates that income has no or weak causal effects on health (33).

Our analysis of the evolution of the relationship between real income and life expectancy (the so-called Preston curve relation) further supports the view that the income gradient, as observed in cross-sectional associations between income and life expectancy, is not the primary driver behind the emergence of this gradient. Instead, there is a clear “shift” in the relationship between income and life expectancy.

Our cause of death analysis builds on earlier research investigating causes of death to explain the widening income or SES gradient in life expectancy. Historical studies from Swedish regions suggest that cause-specific gradients for cardiovascular disease and cancer began to emerge around 1970. Higher socioeconomic groups benefited earlier from these declines (3, 34). Consistent with these findings, we find that reductions in mortality from circulatory diseases accounted for the largest overall gains in life expectancy across both the highest and lowest income quartiles. However, the differential gains between the highest and lowest income quartiles were larger for cancers for women and of similar size as circulatory diseases for men.

The decomposition of the income gradient by causes of death further reveals that a significant portion of the increase is attributable to preventable causes, likely linked to the differential adoption of new life habits. These findings are qualitatively consistent with Nordic studies using more contemporary data, although our broader definition of preventable mortality complicates direct comparisons of magnitudes. Specifically, these studies underscore cardiovascular diseases, along with alcohol- and smoking-related mortality, as key contributors to the income gradient in mortality, with slower declines in mortality observed among lower-income groups since the 1990s (10, 12, 26, 32, 35–37). During 1995–2007, alcohol- and smoking-related deaths accounted for 30 to 50% of the life expectancy gap in Nordic countries (38). More recent Finnish data, however, suggest that while these causes still account for roughly 40% of the overall gap, their role in the recent widening is minimal, with stagnation in the lowest income quintile linked to mortality across a broader range of causes (32). The relative importance of deaths attributable to excessive alcohol consumption and smoking-related mortality has increased as a key driver of mortality inequalities also in other European countries (39–41).

Further emphasizing the role of lifestyle factors in the widening income gradient in life expectancy is the increasing socioeconomic disparity in smoking behavior over time. For Sweden, data from the Level of Living Survey highlight this widening gradient: The odds ratio (95% CI) of smoking between the lowest and highest income quintiles was 0.81 (0.726, 0.894) for men and 0.32 (0.242, 0.398) for women in 1968. In 2017, these figures had increased dramatically to 4.62 (3.189, 6.051) for men and 2.85 (2.046, 3.654) for women (42, 43).

From a policy perspective, the results of this study suggest that reducing income inequality alone may not effectively address life expectancy disparities. The increasing gradient in preventable deaths underscores the disparities in lifestyle improvements, where higher-income groups have been more successful in adopting behavioral changes that improve health, such as smoking, alcohol consumption, and physical exercise. Thus, policies should focus on promoting healthier lifestyles, particularly among lower-income groups (44). However, as noted in recent research, investments in public health targeting older populations can inadvertently increase inequality if lower income individuals are less likely to survive and benefit from such interventions (45).

Finally, the increasing income gradient in life expectancy also has implications for pension systems, as it indicates an increased redistribution from low- to high-income earners, undermining progressive benefit structures. Income-based benefit adjustments or targeted support for lower income retirees could help mitigate these regressive effects (11, 46).

## 3. Materials and Methods

The study was approved by the Central Ethical Review Board (Etikprövningsmyndigheten), reference number 2015/420. Participant consent was waived as the analysis was based on existing anonymized data from four Swedish national registries.

### 3.1. Data Sources and Study Population.

This study analyzed individual-level data from the Population Register, National Tax Register, Longitudinal Database for Health, Insurance and Labour Market Studies (LISA), and the Cause of Death Register. We used income data for the period 1960–2019 and mortality data for 1962–2021 to analyze inequality measures during 1962–2021 for men and 1970–2021 for women.

Income information from 1960 to 2019 was sourced from the National Tax Register, excluding 1967. For 1960–1966, a sample 10% of the tax-filing population was available, and household income was calculated by totaling individual and spousal income. Due to joint taxation prior to 1971, these early data mostly contain the male household head for married couples and are therefore not representative for women. For 1968–2019, the full tax-filing population was available, and household income was calculated by combining complete taxpayer income records with household identifiers from the Population Register and the LISA database, defined pre-1987 as married couples and post-1987 as married couples or cohabiting couples with joint children. The analysis excluded individuals with missing, negative, or zero income and the lowest 3% of positive earners (around 7% of the sample in recent years).

Mortality data from 1962 to 2021 came from the Cause of Death Register, including the date of death of all individuals aged 40 y or older, without adjusting for ethnic composition [unlike (8)]. Sensitivity tests excluding first-generation immigrants showed very similar results (*SI Appendix*, Fig. S2).

The population consisted of 9,395,993 people aged 40 y or older and 213,738,123 person-year observations, accounting for 3,874,308 observed deaths. The mean (SD) age in this population was 60.04 (13.19) years in 2021.

### 3.2. Income Measures.

The primary income measure, “equivalised household income,” was derived from the taxable income of individuals, including wages, business profits, pensions, and taxable transfers. This measure was obtained by dividing the household’s total income by the square root of the number of household members. Several components, such as income from real wealth (e.g. owner-occupied housing) and the value of home production/leisure time were not included in the income measure. Households were defined as a single adult or a married or cohabiting couple, with adult children considered separate single adults. Children aged 18 y or under living with their parents were excluded due to inconsistent historical data. Incomes were inflation adjusted to correspond to the price level in 2018 using the CPI. Sensitivity analyses using other income measures, such as individual or disposable income, confirmed the robustness of the main findings (*SI Appendix*, Fig. S2).

Historically, significant gender disparities in labor force participation were evident in the cohorts studied, with women’s participation considerably lower than men’s. For instance, the labor force participation rate of married women rose from 49.1% in 1967 to 83.5% in 1980 (47) and has continued to increase (48). Consequently, individual income often fails to accurately reflect women’s living standards, particularly for those married or cohabiting with higher-income partners. *SI Appendix*, Fig. S2 compares income gradients for men and women across various household-level income measures and an individual-level measure of taxable income. The findings show that women’s income gradients are flatter when individual taxable income is used. We therefore adopt equivalised household income as our preferred income variable, as it provides a more reliable proxy for material living standards, especially for women.

### 3.3. Causes of Death.

The year and cause of death were obtained from the Cause of Death Register, with causes classified into subgroups using the International Classification of Diseases ICD-6 through ICD-10 chapters.

Deaths were first categorized by main causes of death by ICD chapter (*SI Appendix*, Table S1), separating between Circulatory diseases, Cancers, External causes, and Other causes.

Deaths were then classified by avoidability following the OECD/Eurostat list (49) and matched to respective ICD codes (*SI Appendix*, Table S2). Deaths were identified as Treatable, Preventable, Both treatable and preventable, or Nonavoidable. Preventable mortality is caused by health conditions that can be avoided through effective public health and primary prevention interventions. Treatable mortality is caused by health conditions avoidable through timely and effective healthcare interventions, including secondary prevention and treatment to lower mortality rates after disease onset. A death was classified as preventable or treatable if at least one primary or secondary cause of death fell into the respective category.

### 3.4. Statistical Methods.

This study followed previous research (8, 9) methodology with minor adaptations necessary to accommodate data from the early decades to obtain income ranks. Individuals received percentile ranks (1 to 100) based on equivalised household income by gender, age, and year. For specific analyses, incomes were grouped into quartiles or ventiles. For the rank calculation, income from two years (three years in 1969 due to missing data in 1967) prior to calculating within-group mortality rates was used. Due to using income from two years prior and incomplete income data for women until 1968 (Section 3.1), ranks and thereby inequality measures were calculated from 1962 to 2021 for men and from 1970 to 2021 for women.

The approach diverges from previous research (8) that used the income rank at 63 y of age for older ages, due to data constraints in the 1960s and 1970s. Sensitivity checks using the ranking approach in previous research (8) confirmed that the main findings were not affected by this deviation, likely because the income measure includes pension income.

Period life expectancy at age 40 was calculated using annual mortality rates by sex, age, and income percentile, assuming constant future mortality rates. To counteract unreliable mortality estimates in smaller percentile groups, the study applied the Gompertz–Makeham law, positing a linear link between age and log mortality for ages 40 to 76 and extrapolating mortality rates for ages 77 to 89. For ages 90 to 100, Sweden’s overall mortality rates from Statistics Sweden were used, overlooking income percentile mortality variances. Life expectancy calculations used the integrated survival function from these rates (*SI Appendix*, Fig. S1).

To calculate the years of life expectancy gained from reduced mortality across various causes of death, as shown in Fig. 4, we apply a model from previous research (24) and proceed as follows: We take the average mortality rates over three years in the beginning and end of the observation period and calculate the difference between 1962–64 and 2019–21 for men and 1970–72 and 2019–21 for women by causes of death, divided by the sum of differences among all causes and multiplied by the increase in life expectancy between ages 40 to 74 over the period. The exact numbers are presented in *SI Appendix*, Table S3.

*SI Appendix*, Figs. S3 and S4 presents a comparison of the income gradient in life expectancy in Sweden with findings from Norway (9), the United States (8), and Canada (16). To ensure consistency across these studies, several methodological adjustments were made to align the income measures and sample restrictions, which are described in detail along with the results in that section.

Since our main results are obtained for the entire Swedish population and are not based on any statistical model, we do not report any CIs or SEs, other than for the results on average income gradients reported in Section 1.1 which are estimates from regression models and the results referred to in Section 2 that are obtained from surveys.

The results were obtained using Stata 17.0, MP-Parallel Edition (StataCorp).

## Supplementary Material

Appendix 01 (PDF)

## Data Availability

Data cannot be shared: The paper included in this submission uses very rich register-based individual data. There are restrictions concerning the distribution of these data. It means that we will be unable to meet all aspects of the data availability requirements. First, the data are not proprietary in the sense that we have exclusive access to them. Any researcher can obtain them through Statistics Sweden subject to the same conditions as us. Furthermore, those wishing to perform replication analyses can apply for the data by contacting us on ifau@ifau.uu.se. The researcher will be granted access to the data to the extent necessary to perform replication provided he/she signs a confidentiality agreement saying that the data will only be used for the stated purposes and not transferred to any third party. The practical arrangements for accessing the data will to some extent depend on the location of the researcher and must be handled on a case-by-case basis.
