# Supplementary material for: The rising income gradient in life expectancy in Sweden over six decades
Source: Proc Natl Acad Sci U S A. 2025 Mar 31;122(14):e2418145122. doi: 10.1073/pnas.2418145122 (PMC12002178; doi:10.1073/pnas.2418145122)
Supplement: Supplementary file 1 — Appendix 01 (PDF) [file pnas.2418145122.sapp.pdf]

Supporting Information for

The rising income gradient in life expectancy in Sweden over six decades

Johannes Hagen, Lisa Laun, Charlotte Lucke, Mårten Palme

Mårten Palme.
E-mail: marten.palme@su.se

This PDF file includes:

Supporting text
Figs. S1 to S7
Tables S1 to S3
SI References

Supporting Information Text

1. Data

List of data sources:

1. Population data: The population data list everyone registered in Sweden during the years of interest with information of birth year, birth country, etc.

2. Income and tax registers:

    - Taxable Income (*Inkomster*) 1960–1966: This dataset contains data on income, marital status, occupation, and spousal income. Including everyone born on the 5th, 15th, and 25th of each month results in a 10% random sample of tax filers. Due to joint taxation of couples until 1971, many women are missing, and the sample is not representative for them.

    - National Tax Register (*Inkomst- och taxeringsregistret, IoT*) for the years 1968–2021: This database contains many income measures, along with personal information like marital status and household affiliation for all Swedish tax filers.

3. Longitudinal Database for Health, Insurance and Labour Market Studies (LISA): This register consists of data on various income sources and household identifiers for the period 1990–2021.

4. National Cause of Death Register: This register contains the date of death for all permanent residents in Sweden since 1961, with details on the cause of death by ICD chapter and treatable and preventable indicators.

5. Aggregate age-specific mortality rates from Statistics Sweden.

2. Income Measures

Figure S2 shows the income gradients when using different income measures for men and women, respectively. All measures show a clear increase for men, with the individual measures being slightly higher than the rest. The income gradient grew larger after 1980 for women.

3. International Comparison

In this section, we compare the mortality gradient in Sweden to estimates for the US in Chetty et al. (1) and the comparative estimates for Norway in Kinge et al. (2) and for Canada in Milligan and Schirle (3).

Some methodological adjustments were necessary for consistency with Chetty et al. (1) and Kinge et al. (2). Most importantly, a different income measure was used, including all earnings from labour and work-related benefits such as unemployment, parental leave and sickness benefits, but excluding disability insurance and early retirement benefits. The same sample restrictions as in Chetty at al(1) were applied, meaning dropping zero income persons and the bottom 2%. Using the new income measure resulted in dropping more individuals with disability insurance benefits or pensions as their main source of income compared to our main analysis. The selection of samples by Chetty et al. (1) essentially implies the exclusion of individuals outside the labor force. During the 2001–2014 period, the average LFP rate in ages 20–65 was around 78 percent in the US, 82 percent in Norway and 84 percent in Sweden. In addition, household income was not adjusted for household size. Income ranks were assigned similarly, but for those above age 63, ranks from age 63 were kept, which did not pose an issue when limiting the analysis to the 2001–2014 period.

Chetty et al. (1) noted a 14.6-year gain in life expectancy at age 40 for US men (10.1 years for women) from the first to the 100th percentile in the income distribution during 2001–2014. Using the same time period and consistent income concepts and methodologies, Norway's data showed a 12.9-year increase for men and 7.4 for women (2). The corresponding figures for Sweden were 6.17 for men and 4.2 for women (see Figure S3).

These gaps were significantly smaller in Sweden than those observed in the US and Norway, mainly due to higher life expectancies in Sweden among the lowest 10 percentiles. However, the results were sensitive to the precise definition of the income measure used. Although the income measure used in the main part of the study indicated larger gaps in life expectancy, the income measure in this comparison produced a more linear gradient, as seen in Figure S3. As pointed out in a note by Cutler (4), the challenges of aligning income concepts and sample restrictions across countries underline the importance of caution when making international comparisons of income gradients in life expectancy and their implications for the relationship between income and health.

Canadian estimates in ventiles by Milligan and Schirle (3), on the other hand, seen in Figure S4, closely mirrored the ones for Sweden when the same methods were applied.

4. Cause of Death

Table S1 shows the aggregation of ICD-codes into chapters. Circulatory diseases and nervous system disorders had to be aggregated into one category, because there was an important reclassification when the system changed from ICD7 to ICD8. *Vascular lesions affecting central nervous system* (330–334), classified as disorders of the nervous system in ICD7, were moved to the subsection *Cerebrovascular disease* (430–438) of the chapter on circulatory diseases in ICD8. This resulted in a sharp drop in deaths from nervous system disorders from approximately 11% to 1% and a sharp increase in deaths from circulatory diseases from approximately 39% to 49%. Counting this category as a circulatory condition, it follows that approximately 3% of deaths in the aggregated category in the 1970s and 1980s are due to other disorders of the nervous system, while circulatory diseases account for the remaining 97%. In the 1990s, deaths from circulatory diseases started to decrease rapidly and deaths from nervous system disorders increased, such that the share of nervous diseases in the aggregated category rose to 17% at the end of the 2010s.

Table S2 relies on the OECD list of treatable and preventable diseases (5). The mapping across ICD-9–ICD-6 was done by the authors and is an approximation, due to difficulties to perfectly match the codes across time. Using these tables, the National Board of Health and Welfare coded indicators for preventable and treatable diseases from the National Cause of Death Register. A death was classified as preventable/treatable if at least one cause of death (primary or secondary) was preventable/treatable.

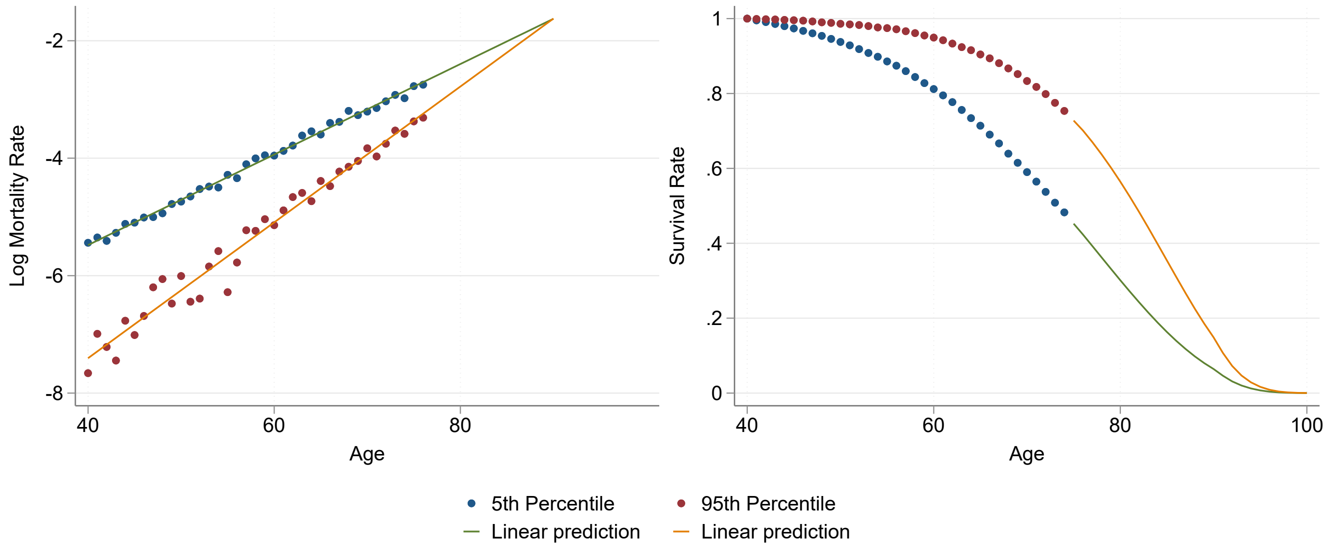

Fig. S1. Mortality rates and survival rates for men in Sweden, 1962–2021. The left panel presents the log-linear mortality approximation by age for the 5th and 95th percentiles in the income distribution. The right panel illustrates the resulting survival functions for each of the two groups based on real data up to age 76 and predictions beyond that age. Estimates were derived from the male Swedish population aged 40–76, excluding individuals with zero, negative or missing income and the lowest 3% incomes.

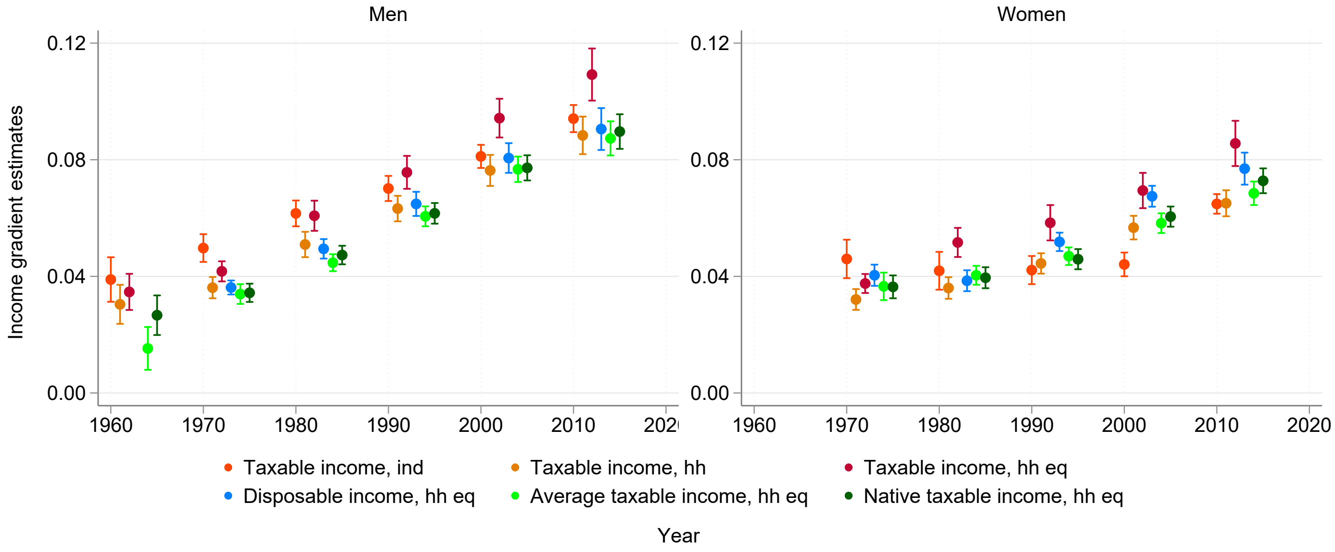

Fig. S2. Comparison of income gradients for different income measures for men and women. These income measures include taxable income at the individual level ("ind"), household level ("hh"), and equivalised household level ("hh eq"). It also includes disposable income on equivalised household level ("Disposable income, hh eq"), average equivalised household taxable income over the past five years ("Average taxable income, hh eq") and household taxable income excluding immigrants ("Native taxable income, hh eq").

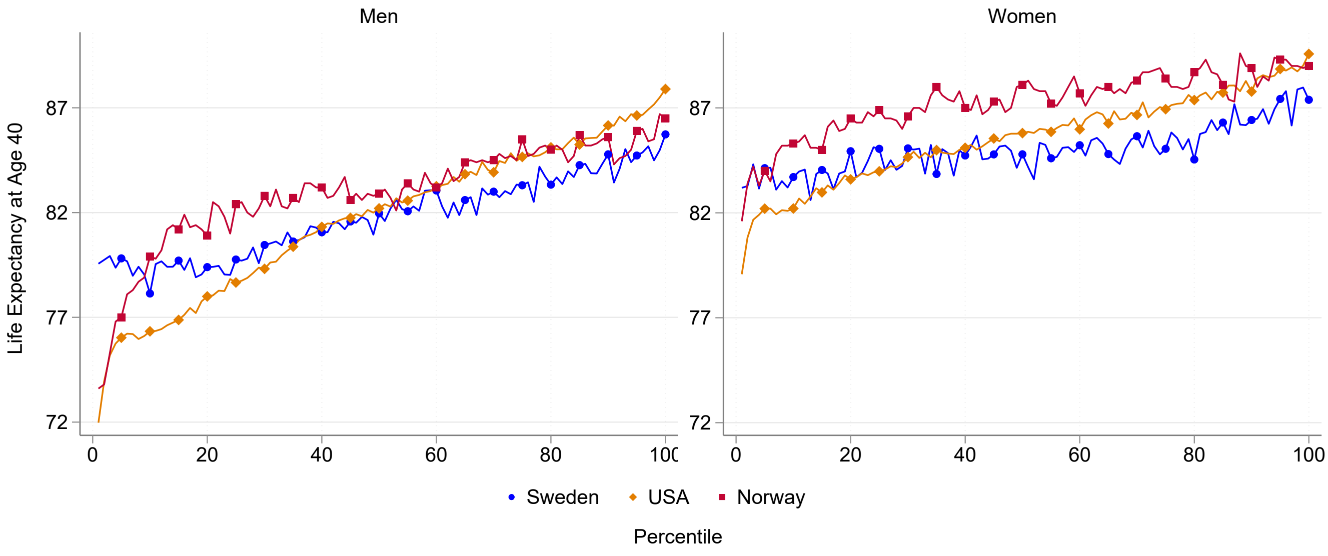

Fig. S3. Comparison to US and Norway, 2001–2014, for men and women. Life expectancy at age 40 by income percentile.

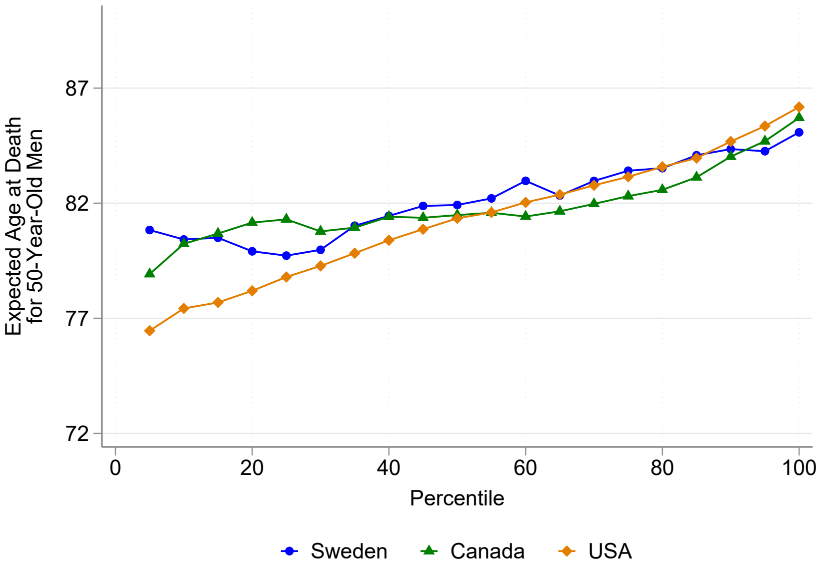

Fig. S4. Comparison to US and Canada in ventiles, 2001–2014, for men. Life expectancy at age 50 by income percentile.

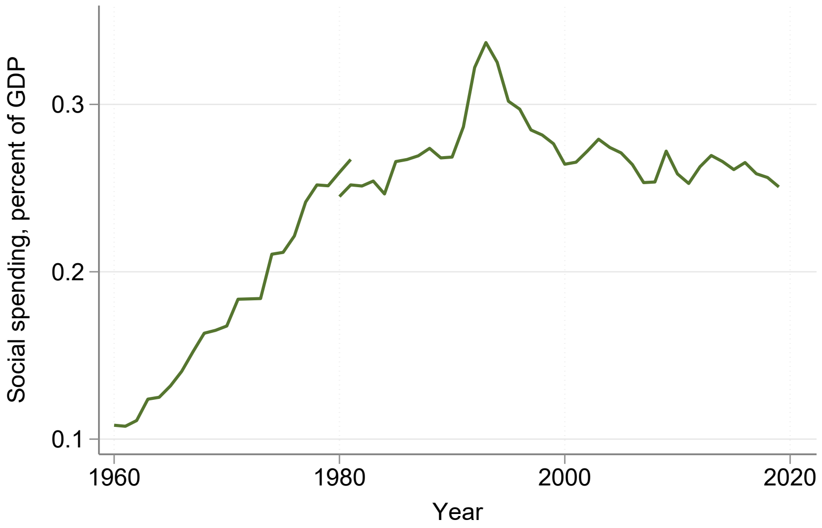

Fig. S5. Social spending as a percentage of GDP in Sweden, 1960–2019. Until 1981, the data was compiled from OECD sources by Lindert (6). A different time series from the OECD database was used from 1980.

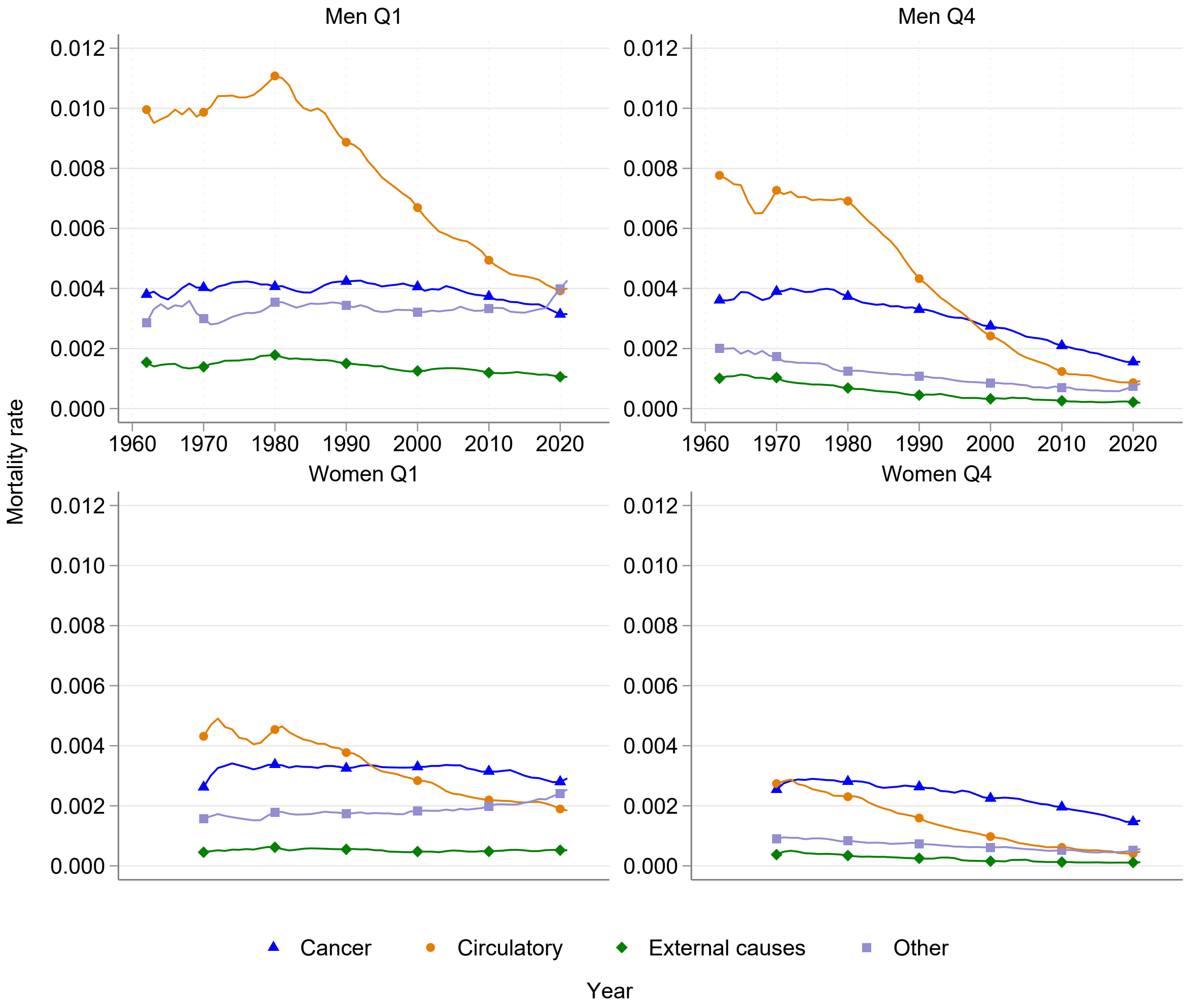

Fig. S6. Mortalities by main ICD chapter, 1960–2021, for men and women in the 1st and 4th income quartiles.

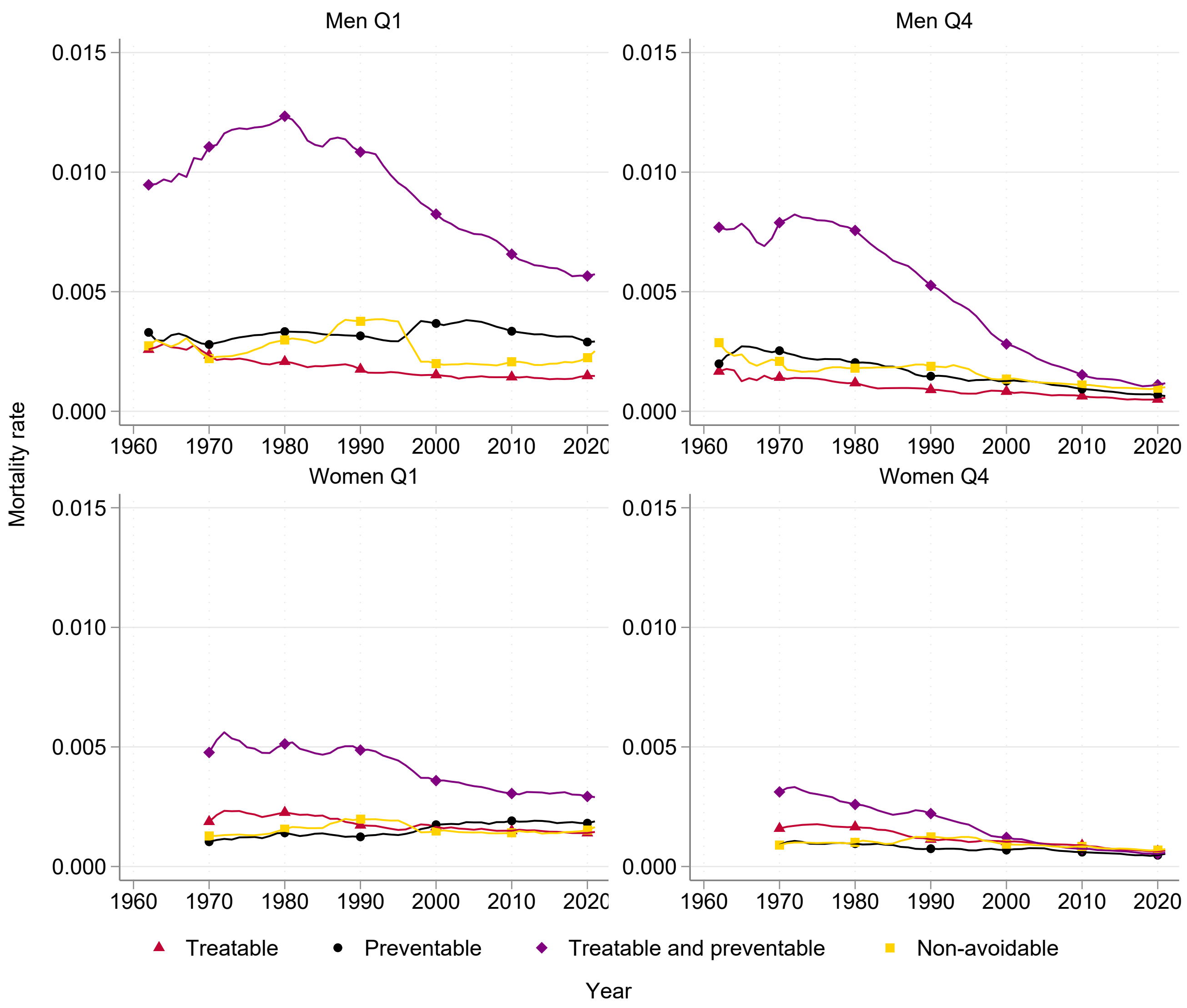

Fig. S7. Mortalities by main avoidability, 1960–2021, for men and women in the 1st and 4th income quartiles.

Table S1. Cause of Death Aggregation by Chapter

Cause of death	ICD-10	ICD-9	ICD-8	ICD-7 & -6
**Infectious diseases**	A00–B99	001–139	000–136	001–138
**Cancer**	C00–D49	140–239	140–239	140–239
**Mental disorders**	F00–F99	290–319	290–315	300–326
**Circulatory diseases**	G00–G99, I00–I99	320–459	320–458	330–468
**Respiratory diseases**	J00–J99	460–519	460–519	470–527
**External causes**	V01–Y99	800–999	E800–E999	E800–E999
**Other diseases**	D50–E89, H00–H95, K00–R99	240–289, 520–799	240–289, 520–796	240–299, 530–795

Notes: Circulatory diseases and nervous system disorders were aggregated into one category due to reclassification when the system changed from ICD7 to ICD8. In the main analysis, we combine Infectious diseases, Mental disorders, Respiratory diseases and Other diseases into the category "Others".

Table S2. Mapping of avoidable conditions across ICD 6–10

**ICD-10**	**ICD-9**	**ICD-8**	**ICD-7 & -6**
Preventable conditions			
A00–A09 A15–A19 A33–A37 A39 A403 A413 A492 A50–A60 A63–A64 A80 B01 B05–B06 B15–B24 B50–B54 B90 C00–C16 C22 C33–C34 C43 C45 C53 C67 D50–D53 E10–E14 E244 F10–F16 F18– F19 G000–G001 G312 G621 G721 I10–I13 I15 I20–I25 I60–I71 I739 J09–J11 J13–J14 J40–J44 J60–J70 J82 J92 K292 K70 K73 K740–K742 K746 K852 K860 Q00–Q01 Q05 Q860 R780 V01–Y34	001–018 032–033 036–037 0380 03841 0415 042–045 052 055–056 070 7811 084 090–099 137 140–151 155 162–163 172 180 188 2262 249–250 280–281 3200–3201 3204 401– 405 410–414 430–438 440–441 481 4822 4823 487 490–492 500–508 5183 7713 740–741 7420 800–999	001–019 032–033 036 03800 040–043 052 055–056 070 07399 084 090–099 140–151 155–156 162–163 172 180 188 250 280–281 3200–3201 400–404 410–414 430–438 440– 441 470–474 4821–4822 490–492 500–508 740–741 E800–E999	001–019 020–039 040–049 051 05300 055–057 061 064 080–081 085–087 092 110–117 140–148 150–151 155–156 162 163 171 181 190 260 291–293 307 322–323 330–334 340 420 440–447 450–451 453 480–483 501–502 523–525 527 753 E800–E999
Treatable conditions			
A15–A19 A38 A400–A402 A404–A409 A410–A412 A414–A419 A46 A481 A491 B90 C18–C21 C50 C53–C55 C62 C73 C81 C910–C911 D10–D36 E00–E07 E10–E14 E240–E243 E248–E249 E27 G002–G003 G008–G009 G03 G40–G41 I00–I13 I15 I20–I26 I60–I69 I70–I71 I739 I80 I829 J00–J06 J12 J15–J18 J20–J22 J30–J39 J45–J47 J65 J80 J81 J85–J86 J90 J93–J94 K25–K28 K35–K38 K40– K46 K80–K83 K850–K851 K853–K859 K860–K861 K863–K869 L03 N00–N07 N13 N17–N21 N23 N25–N27 N341 N35 N40 N70– N73 N750–N751 N764 N766 O00–P96 Q20–Q28 Y40–Y84	010–018 0341 035 0381–0383 03840 03842–03849 0388–0389 0479 137 180 153–154 174 179 182 186 193 201 204 210–229 240–246 249–250 255 320–322 345 390–398 401–405 410–415 430–438 440–441 451 4539 460–466 470–478 480 4820–4821 4823–4824 4828–4829 483–486 493–494 51882 510–514 53084 531–534 540–543 550–553 574–577 580–589 591–592 59780 598 600 614–616 630–679 681–682 745–747 760–779 7880 9940 99591 99709 E860–E879 E930–E949	010–019 0340–0341 0381–0389 035 045–046 153–154 174 180 182– 183 186 193 201 204 210–229 240–256 320 345 390–398 400–404 410–414 426 430–438 440–441 451 4539 460–466 480–481 48201 4822 48230 483–486 493 500–508 510–514 518 531 534 540–543 550– 553 573–574 576–577 580–586 591–592 597–598 600 612–613 616 620 622 625 630–678 681–682 745–747 760–779 78602 E850–E859 E930–E936 E947 E949	001–019 050–052 05310–05349 154 170–173 178 194 201 20400 20455 210–229 241 250–254 260 274 330–334 340–341 353 400–416 420 440–447 450–451 453–454 463–466 470–475 490–493 500 510–522 526 540–545 550–553 560–561 584–585 587 590–594 600–604 608 610 622–624 626 630 63711 640–689 691–693 754 78550 E940–E959

Notes: A death was classified as preventable/treatable if at least one cause of death (primary or secondary) was preventable/treatable.

Table S3. Contribution of each cause of death to the overall life expectancy increase for men and women (in years)

	Men 1962/64–2019/21			Women 1970/72–2019/21		
	**Quartile 1**	**Quartile 4**	**Difference Q4–Q1**	**Quartile 1**	**Quartile 4**	**Difference Q4–Q1**
**Main Cause of Death**						
Cancer	0.1876	0.5577	0.3701	0.0585	0.3745	0.3160
Circulatory diseases	1.4292	1.8548	0.4256	0.8540	0.7122	–0.1418
External causes	0.0875	0.2267	0.1392	–0.0092	0.1104	0.1196
Other causes	–0.1979	0.3353	0.5332	–0.2378	0.1300	0.3678
**Avoidable mortality**						
Treatable	0.3074	0.3462	0.0388	0.2314	0.2945	0.0631
Preventable	0.0180	0.4473	0.4293	–0.2162	0.1583	0.3745
Treatable and preventable	0.9917	1.7650	0.7732	0.7249	0.7908	0.0660
Non-avoidable	0.1893	0.4160	0.2267	–0.0745	0.0834	0.1580
**Total**	1.5064	2.9745	1.4681	0.6656	1.3271	0.6616

Notes: Data from the total Swedish population aged 40–74, excluding individuals with missing, negative or zero income and the lowest 3% income. The table shows the difference in mortality rates between 1962/64–2019/21 for men and 1970/72–2019/21 for women by cause of death, divided by the sum of differences among all causes of death and multiplied by the increase in life expectancy between ages 40–74 over the same periods.

References

1. R Chetty, et al., The Association Between Income and Life Expectancy in the United States, 2001–2014. *JAMA* **315**, 1750–1766 (2016).
2. JM Kinge, et al., Association of household income with life expectancy and cause-specific mortality in Norway, 2005–2015. *JAMA* **321**, 1916–1925 (2019).
3. K Milligan, T Schirle, The evolution of longevity: Evidence from Canada. *Can. J. Econ. canadienne d'économique* **54**, 164–192 (2021).
4. DM Cutler, Life and death in Norway and the United States. *JAMA* **321**, 1877–1879 (2019).
5. OECD/Eurostat., Avoidable mortality: OECD/Eurostat lists of preventable and treatable causes of death, (Organisation for Economics Co-operation and Development), Technical report (2019).
6. PH Lindert, *Growing public: Social spending and economic growth since the eighteenth century.* (Cambridge University Press), (2004).
